# Cross-Sectional Association between Length of Incarceration and Selected Risk Factors for Non-Communicable Chronic Diseases in Two Male Prisons of Mexico City

**DOI:** 10.1371/journal.pone.0138063

**Published:** 2015-09-18

**Authors:** Omar Silverman-Retana, Ruy Lopez-Ridaura, Edson Servan-Mori, Sergio Bautista-Arredondo, Stefano M. Bertozzi

**Affiliations:** 1 Center for Health Systems Research, National Institute of Public Health, Cuernavaca, Mexico; 2 Center for Population Health Research, National Institute of Public Health, Mexico City, Mexico; 3 School of Public Health. University of California, Berkeley, California, United States of America; The University of Tokyo, JAPAN

## Abstract

**Background:**

Mexico City prisons are characterized by overcrowded facilities and poor living conditions for housed prisoners. Chronic disease profile is characterized by low prevalence of self reported hypertension (2.5%) and diabetes (1.8%) compared to general population; 9.5% of male inmates were obese. There is limited evidence regarding on the exposure to prison environment over prisoner’s health status; particularly, on cardiovascular disease risk factors. The objective of this study is to assess the relationship between length of incarceration and selected risk factors for non-communicable chronic diseases (NCDs).

**Methods and Findings:**

We performed a cross-sectional analysis using data from two large male prisons in Mexico City (n = 14,086). Using quantile regression models we assessed the relationship between length of incarceration and selected risk factors for NCDs; stratified analysis by age at admission to prison was performed. We found a significant negative trend in BMI and WC across incarceration length quintiles. BP had a significant positive trend with a percentage change increase around 5% mmHg. The greatest increase in systolic blood pressure was observed in the older age at admission group.

**Conclusions:**

This analysis provides insight into the relationship between length of incarceration and four selected risk factors for NCDs; screening for high blood pressure should be guarantee in order to identify at risk individuals and linked to the prison’s health facility. It is important to assess prison environment features to approach potential risk for developing NCDs in this context.

## Introduction

Although infectious diseases and mental health issues have been the main areas of research related to health and risk behaviors among prisoners,[1,2] interest in exploring other health problems is increasing. A recent systematic review revealed that the prevalence rates of risk factors for non-communicable chronic diseases (NCDs) among inmates varies widely.[3] Overweight and obesity prevalence rates in prisons in high income countries like in Australia, the United Kingdom and the United States are higher than in prisons in low income countries like Bangladesh, Cambodia and Nigeria. However, compared to the general population, prevalence rates of obesity and overweight are higher in prisoners in high income countries; whereas in low income countries these differences are in the opposite direction.[3] Furthermore, prevalence of self-reported hypertension in prisons was 14% in Australia, close to 5% in England, Wales and Ireland, and up to 30% in US prisons;[4] whereas self- reported prevalence of diabetes was less than 5% in Australia, England, Wales and 10% in US prisons.

Regrettably, most of the evidence on these health issues comes from high-income country prisons which might not be applicable in low- and middle-income countries. In Latin America, Brazil has the largest prison population followed by Mexico and Colombia.[5] Within Mexico, Mexico City has the highest incarceration rate. Estimations using recent data from the Mexico City’s Penitentiary System Secretariat and the 2010 Population and Household Census showed an imprisonment rate of 453/100,000 inhabitants. As a result of recent reforms to the penal codes that hardened sentences,[6] Mexico City’s prison population increased from 32,628 to 40,102 inmates from December 2006 to December 2009, exacerbating already existing problems of overcrowding, lack of appropriate access to health services, and other living conditions in the prisons.[7] Particularly, insufficient amount of health personnel to cover prisoner’s health needs is combined with shortage of medications and supplies to deliver health care to large amounts of prisoners.

Despite provision and storage of food complies with prison general food-safety regulations and centrally pre-defined monthly menus; final preparation and distribution of food are prisoner’s tasks. In this sense neither regulation of dietary salt amount (salt added during preparation as well as delivering food with high content of salt) nor distribution of a standard portion size is established. Other factors that influence prisoner’s diet are the family visits; prisoner may acquire food provided by the family. One last consideration is the lack of attention on prisoners with special dietary requirements.[7]

As part of an integrated strategy to evaluate health conditions among prisoners in Mexico City, we recently evaluated the prevalence of communicable diseases and NCDs prevalence.[8] Obesity prevalence was 9.5% in two male prisons, lower than that in the general population. Previous diagnosis of hypertension was reported in 2.5% and diabetes in 1.8% of prisoners. As with obesity, prevalence rates of these self-reported chronic diseases were lower in prisoners than in the general population.[8]

Prisons tend to be unhealthy environments for three principal reasons: 1) composition of the population, in which people with poor health-risk behaviors and undetected or untreated conditions are over-represented,[9] 2) prison exposures,[10] particularly harmful in low- and middle-income countries (drug consumption, violence, overcrowded facilities and the poor quality diet provided by the penitentiary systems) and 3) poor access to low quality health care.[1,2] In contrast to mental health, sexually transmitted infections (STI) and substance abuse, few studies have focused on the potential effect of prison environments on cardiovascular risk factors.[10] The objective of this study is to examine in two male prisons of Mexico City the relationship between length of incarceration, as a proxy for “dose” of exposure to the prison environment, and selected risk factors for NCDs, including: body mass index (BMI), waist circumference (WC) and blood pressure (BP).

## Materials and Methods

### Study design and characteristics of the study population

We analyzed data from a cross-sectional study carried out in two large male prisons from the Mexico City’s Penitentiary System between June and September 2010. Prisoners were invited to participate in a general health assessment including a screening questionnaire for pulmonary tuberculosis and serological screening tests for STI, including: HIV, Hepatitis B, Hepatitis C, and syphilis. Anthropometric measurements, blood pressure assessment and screening for type 2 diabetes were conducted by trained standardized field personnel from the Mexican National Institute of Public Health (INSP). The details of the fieldwork assessment and blood sample tests are detailed elsewhere.[8] The project was reviewed and approved by the Committees of Research, Biosafety and Ethics of the INSP (Research committee: 821 N° 712, Biosecurity committee: 813).

Of 20,176 prisoners, 15,517 decided to participate in the study and complete the initial interview (~77%). For this analysis we excluded prisoners with missing outcome values (NCD risk factors) (n = 103), missing length of incarceration (main exposure variable, n = 36), length of incarceration longer than 10y (outliers, n = 626), age younger than 18y at admission to the prison (main stratification variable, n = 337) and previous diagnosis of diabetes, hypertension and dyslipidemia under treatment (n = 329) to avoid reverse causation on the levels or report of NCD risk factors after the diagnosis of medical condition. Additional analyses were performed to assess the potential underestimation of the association studied, due to excluding previously diagnosed patients. The results show similar estimates as the ones from the models excluding these prisoners.

After all exclusions the final analytical sample included 14,086 prisoners aged 18y and older (for details of the analyzed sample, see S1 Fig). Potential selection bias was evaluated by differences between the sample with complete data (14,086) versus individuals excluded (1,431); as expected for the exclusion criteria (long incarceration rates and previous diagnosis of chronic diseases) prisoners included in the sample were younger, had lower length in prison, lower BMI, WC and BP.

### Outcome variables

Height, weight and waist circumference were measured twice (and averaged) with inmates wearing light clothing after removing shoes, belts and items in pockets. We calculated body mass index as kg/m^2^. Blood pressure was measured twice in both arms using a digital sphygmomanometer, this was repeated with a minimum 2-minute interval; and then all averaged readings of systolic and diastolic blood pressures was used.[11] Cutoff points and definitions for BMI followed the WHO definition (underweight (BMI <18.5), normal (BMI 18.5–24.9, overweight (BMI 25.0–29.9) and obese (BMI ≥30);[12] abdominal obesity (WC>102cm) and metabolic syndrome used the NCEP/ATP III definition (3 out of 5 features);[13] and blood pressure cutoff point used the 2007 Guidelines for the Management of Arterial Hypertension (≥140/90mmHg).[14]

A validated risk factor questionnaire adapted from the original American Diabetes Association (ADA) screening questionnaire for type 2 diabetes used in Mexico was applied during the first visit.[15] High risk inmates for type 2 diabetes or hypertension followed a stepwise diagnostic algorithm, including non-fasting and fasting (confirmatory) blood glucose (for details of the performed algorithm, see S2 Fig). We identified self-reported previously diagnosed inmates with diabetes, hypertension and dyslipidemia. They underwent a standardized testing for fasting blood glucose and lipid profile (total cholesterol-TC, low/high density lipoprotein LDLc/HDLc, and triglycerides-TG) measurements for metabolic assessment. In addition, a random subsample of 5% of inmates independently of baseline risk criteria was selected to estimate the prevalence of metabolic syndrome. All serum samples were analyzed with spectrophotometry in a single central laboratory certified for clinical laboratory services.

### Exposure variable

Length of incarceration was derived from the date of the interview minus the date of admission to the prison for current incarceration. The resulting days of incarceration were then converted into years and divided into incarceration length quintiles. As mentioned previously, prisoners with incarceration lengths over 10y (outliers) were excluded to avoid spurious association explained by extreme values. However, in additional analysis their inclusion did not modify the main conclusions or the results. Our analysis included all inmates without restricting to first time offenders. Given the lack of information on previous incarcerations, a cumulative length of incarceration from previous sentences for recurrent offenders was not considered. Age at admission was stratified into three age groups: <25y, between 25 and 39y, and 40y and older. Prison facility (0 = north, 1 = south) was considered as a potential effect modifier variable and it was also interacted with length of incarceration.

### Statistical analysis

With the idea that unhealthy environments might vary across prison facilities, non-parametric Mann Whitney median test for continuous variables and Chi-square test for categorical variables were performed to explore differences between the two prisons. We evaluated the association between the length of incarceration (in quintiles) and each of the selected risk factors (BMI, WC, systolic (SBP) and diastolic blood pressure (DBP)) using Quantile Regression Models.[16,17] This approach was used as the outcomes analyzed were not normally distributed. All models were adjusted by current age, facility and an interaction term between prison facility and length of incarceration; models for systolic and diastolic blood pressure were additionally adjusted by BMI. Test for trend across length of incarceration quintiles was evaluated using length of incarceration p50 within each quintile category.

To evaluate the potential effect modification of the age of admission (continuous in years) over the association between length of incarceration (continuous in years) and the NCD risk factors, we estimated the same model in each of the three pre-defined age strata at admission. The significance of this effect modification was evaluated by the significance of the interaction term between age at admission and length of incarceration, because we were unable to use the Log Likelihood ratio test in quantile regression models. All statistical analysis was performed using Stata version 13.1.[18]

Database used for this analysis is available on http://dx.doi.org/10.6084/m9.figshare.1496589. Also find the study questionnaire on http://dx.doi.org/10.6084/m9.figshare.1496590 for further consultation and please read the author's note before using data on: http://dx.doi.org/10.6084/m9.figshare.1496593.

## Results

Table 1 shows differences among the two prison facilities in terms of prisoners’ characteristics, selected risk factors, type 2 diabetes screening questionnaire features, and fasting blood assessment (among the 5% random subsample). Current age and age at admission to the prison were similar among prisoners in both facilities; median length of incarceration was higher in the southern facility (1.97y) compared to the northern facility (1.86y). A higher risk profile was observed among prisoners in the southern facility: higher rates of obesity, abdominal obesity, previously unknown hypertension and scoring ≥10 points on the type 2 diabetes risk questionnaire. However the prevalence of metabolic syndrome among the 5% random subsample was 2.91% without differences between prisons.

**Table 1:** Inmates’ general characteristics, selected risk factors and type 2 diabetes risk factor questionnaire characteristics by prison facility.

	South Prison	North Prison	*p-value*
N (%)	5,319 (37.7)	8,767 (62.2)	
	Median [IQR] or %		
General characteristics			
Current age (yrs.)	31.0 [26.0;38.0]	31.0 [26.0;38.0]	0.964
Age at admission to the prison (yrs.)	28.6 [23.2;35.0]	28.7 [23.5;35.6]	0.187
Length of incarceration (yrs.)	1.97 [0.64;4.08]	1.86 [0.69;3.61]	0.044
Selected risk factors			
BMI (kg/m^2^)	24.8 [22.6;27.4]	23.7 [21.6;26.3]	<0.001
Obesity (≥30)	10.9	7.56	<0.001
Waist circumference (cm.)	85.2 [79.2;93.0]	82.5 [76.9;90.0]	<0.001
Abdominal obesity (≥102)	9.33	5.98	<0.001
Systolic blood pressure (mmHg)	116.2 [109.7;124.5]	115.0 [107.5;123.2]	<0.001
Diastolic Blood Pressure (mmHg)	66.7 [61.2;73.0]	67.0 [61.5;72.7]	0.914
Previously unknown hypertension [≥140/90mmHg]	3.91	3.58	0.316
Type 2 diabetes risk factor questionnaire characteristics			
Daily physical activity at least 30 minutes	65.8	58.4	<0.001
Daily consumption of fruit and vegetables	24.7	15.6	<0.001
First degree family with type 2 diabetes	34.1	31.9	0.008
Score ≥ 10 points	27.3	23.1	<0.001
Random subsample, n (%)	451 (5.14)	257 (4.83)	
Fasting Glucose (mg/dl)	88.0 [84.0;94.0]	87.0 [82.0;94.0]	0.064
Probably unknown impaired fasting glucose [100–125 mg/dl]	9.19	9.98	0.512
Probably unknown diabetes (≥126 mg/dl)	2.16	1.00	
Lipid profile			
Total cholesterol (mg/dl)	161.0 [141.0;184.0]	156.0 [137.0;183.0]	0.227
LDLc (mg/dl)	97.0 [80.0;116.0]	92.1 [76.8;115.3]	0.221
HDLc (mg/dl)	41.2 [36.2;45.8]	41.1 [35.9;46.6]	0.825
Triglycerides (mg/dl)	114.0 [79.0;163.0]	122.0 [86.0;167.0]	0.124
Metabolic syndrome (≥3 features)	2.70	3.01	0.838

Quantile regression models for the selected risk factors are shown in Table 2. Significant negative trend was observed in the association of length of incarceration with BMI (p˂0.01) and WC (p˂0.001); however the association for both outcomes seems to be in “U” shape especially for BMI and the absolute magnitude of these trends are meaningless. In contrast, a significant positive trend (p˂0.001) in blood pressure outcomes (systolic and diastolic) was observed, with monotonic increments across length of incarceration quintiles, with the greatest raise in systolic blood pressure, 5.72 in quintile V. Despite these significant trends, median values in all quintiles are still within normal range.

**Table 2:** Quantile regression models for the main outcomes by length of incarceration quintiles.

Quintile	I	II	III	IV	V	*p for trend*
Median	0.16	0.89	1.95	3.52	5.99	
[Min;max]	[0;0.47]	[0.48;1.35]	[1.36;2.56]	[2.57;4.76]	[4.77;10.0]	
*Anthropometric measures*						
BMI (kg/m^2^)	24.2	0.02 [-0.24; 0.29]	-0.61 [-0.87;-0.34]	-0.57 [-0.84;-0.30]	-0.03 [-0.32;0.26]	0.005
Waist circumference (cm.)	84.1	-0.20 [-0.99; 0.58]	-1.78 [-2.56;-1.01]	-2.00 [-2.78;-1.22]	-1.15 [-2.01;-0.29]	<0.001
Systolic blood pressure (mmHg)	113.0	2.34 [1.51; 3.18]	3.56 [2.74;4.38]	4.12 [3.29;4.94]	5.72 [4.81;6.63]	<0.001
Diastolic Blood Pressure (mmHg)	65.6	1.46 [0.81; 2.11]	2.03 [1.39;2.67]	2.04 [1.40;2.68]	3.01 [2.30;3.72]	<0.001

BMI and WC models adjusted by current age, facility and interaction term between length of incarceration and facility. BP models adjusted by age, facility, BMI and interaction term between length of incarceration and facility. Quintile I value is the predicted median value of the quintile, otherwise quintile values (II-V) are beta coefficients. Values in brackets are CI-95%.

**Fig 1:**
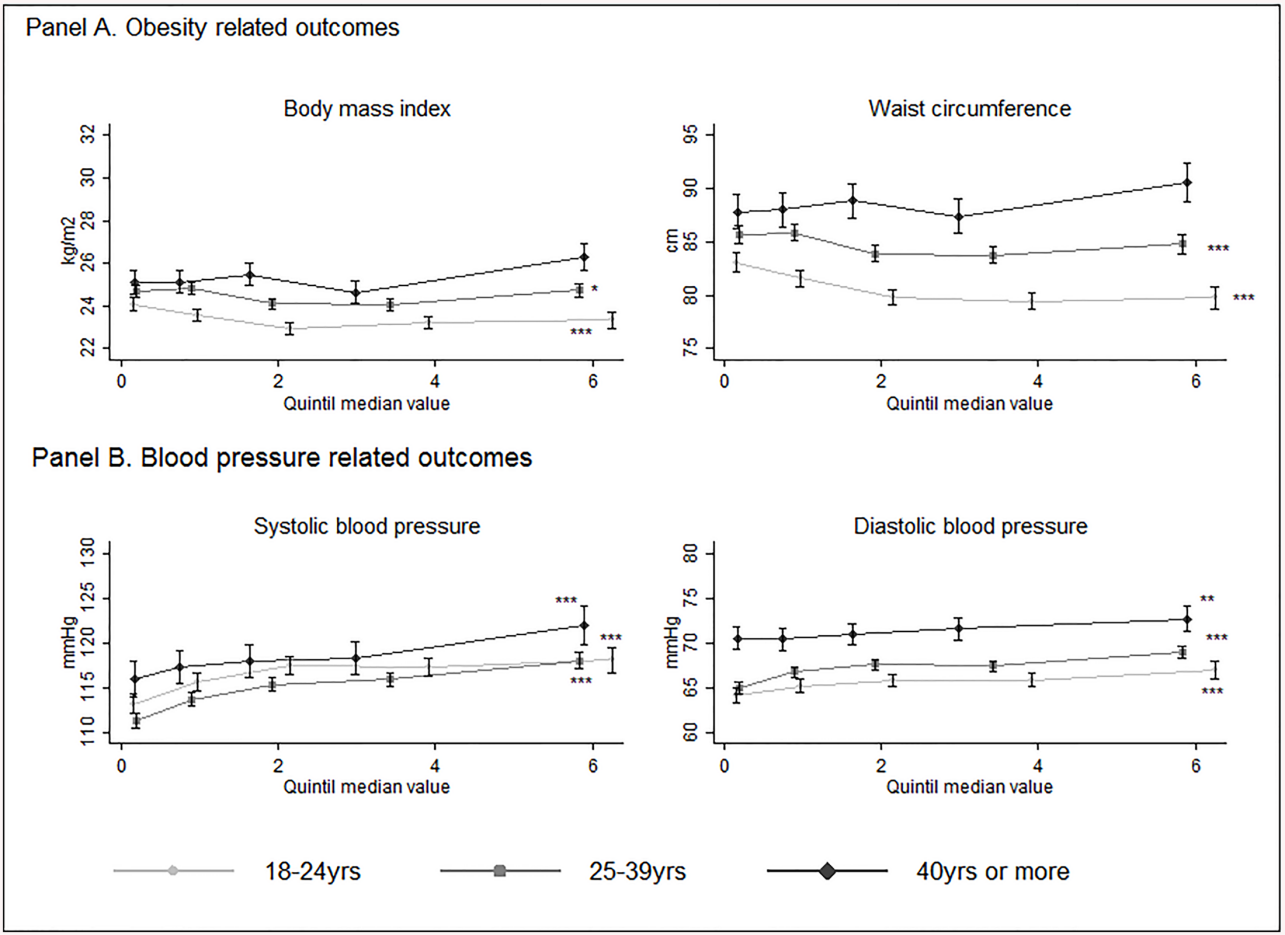
Quantile Regression Estimates for blood pressure and anthropometric outcomes stratified by age at admission to the prison. BMI and WC models adjusted by current age, facility and an interaction term between length and facility. BP models adjusted by age, facility, BMI and an interaction term between length and facility. P for trend: * p ˂ 0.05, ** p ˂ 0.01, *** p ˂ 0.001.

Table 3 shows the median change per year of incarceration for NCD risk factors within each age at admission category after controlling for age (continuously in each age at admission category), facility and an interaction term between length of incarceration and facility. The significant reduction in median predicted value of BMI per year of incarceration was observed among the prisoners who entered the prison at youngest ages particularly in the category of 18–24 year at admission. In the oldest age of admission category this change seems to be positive (increasing both BMI and WC as length of incarcerations increases) however these estimates were significantly different from zero only for WC. In contrast, for the case of systolic and diastolic blood pressure, the median change per year of incarceration was significantly positive and similar in all age at admission categories, with a strongest association among the prisoner who entered at older ages. Fig 1 shows the age at admission stratified associations between length in prison in quintiles and each of the selected NCD risk factors, without assuming lineal association.

**Table 3:** Median change per year of incarceration for the main outcomes; stratified by age at admission to the prison. (Quantile regression model estimates).

Age at admission (yrs.)	Body mass index	P value	Waist circumference	P value	Systolic Blood pressure	P value	Diastolic blood pressure	P value
18–24 (n = 4,597)	-0.12[-0.20;-0.04]	0.004	-0.43 [-0.65;-0.21]	<0.001	0.73 [0.46;1.00]	<0.001	0.39 [0.19;0.60]	<0.001
25–39 (n = 7,584)	-0.001 [-0.06; 0.06]	0.973	-0.13 [-0.31;0.05]	0.158	1.07 [0.89;1.24]	<0.001	0.55 [0.42;0.69]	<0.001
≥40 (n = 1,905)	0.12 [0.00;0.24]	0.034	0.41 [0.04;0.77]	0.027	1.01 [0.58;1.45]	<0.001	0.39 [0.11;0.66]	0.006
Length in prison X age at admission		0.032		<0.001		0.004		0.009
All inmates (n = 14,086)	-0.02 [-0.06; 0.02]	0.332	-0.14 [-0.26;-0.02]	0.017	0.85 [0.72;0.98]	<0.001	0.41 [0.31, 0.51]	<0.001

BMI and WC models adjusted by current age, facility and interaction term between length of incarceration and facility. BP models adjusted by age, facility, BMI and interaction term between length of incarceration and facility.

## Discussion

The main purpose of this cross sectional analysis was to assess the association between length of incarceration and selected risk factors for NCDs (BMI, WC, SBP, and DBP). This study showed original and robust evidence suggesting that length of incarceration is associated with an estimated 4.81% increase in systolic and 4.38% in diastolic blood pressure between quintile I and V. Additionally, length of incarceration was associated with an overall minor decreasing trend in BMI and WC however, especially for BMI, this association seems to be more curvilinear in U shape, with the lowest values in the third quintile of length of incarceration. Stratified analysis by age at admission to the prison, showed the same negative trend in both BMI and WC, except for the older age at admission groups which had a marginal non significant increase. The positive associations between length in prison and blood pressure indicators are significant in all age at admission categories.

Our findings are consistent with Massoglia’s study that found an association between reporting hypertension and longer stays in prison.[10] Possible explanations of the blood pressure findings could be related to longer exposure to stressful environments, high drug consumption (especially those related to cardiovascular outcomes such as cocaine),[19] alcohol consumption,[20] salt consumption,[21] smoking,[22] or other risk behaviors. Nevertheless evidence of the association between length of incarceration and NCDs is scarce. Our results support the hypothesis that exposure to prison environments may have negative consequences on inmates health status.[1,2,9]

In the case of obesity indicators, the association seems to be on the opposite directions; i.e. the prison environment seems to be associated with weight reduction especially during the initial years after admission and among those prisoners who enter prison at younger ages. Although this association might be explained by adopting a healthier behavior in prison through increasing physical activity and it can also be explained by a negative environment characterized by drug abuse or lack of social support. Unfortunately behavior variables were not available for this analysis to enable us to disentangle these competing hypotheses.

Some limitations of the study were the fact that the study relied on a population census that was not designed to explicitly measure the effect of exposure to incarceration (length). Our capacity to make causal inferences is limited by the cross-sectional design of the study. There is a potential selection bias due to non-response rate; unfortunately we could not perform a differences analysis between participants and non-participants, because the profile of the non- participants remains unknown. The estimations may be biased because of unmeasured confounders, such as socio-economic status and the number of times being incarcerated (times exposed). Despite the aforementioned limitations, we believe our study had both internal and external validity at least of similar prison environment in Mexico. Furthermore, the sample size and validity of the exposure variable and the systematic and standardized evaluation of the NCD risk factors are the major strengths of our study.

## Conclusions

This analysis provides insight into the relationship between length of incarceration and four risk factors for NCDs in young male prisoners from two large prisons in the Mexico City Penitentiary System. The positive relationship between these two and the prevalence of undiagnosed NCDs in this population speak to the importance of screening prisoners both at entry and during their incarceration. Although with limited causal inference, the association suggests that incarceration may contribute to development of NCDs. Further studies should explore ways in which the environment could be modified to reduce these risks. There are very few environments as controlled as that of the prison, so it is appropriate to ask: “if we can’t control environmental determinants there, then where?”

## Supporting Information

S1 Fig Participants exclusion criteria flow chart for the final sample analyzed. (EPS)

S2 Fig Screening algorithm flow chart for high risk individuals for type 2 diabetes, elevated blood presure; previously diagnosed inmates and metabolic syndrome features. (EPS)
